# Forgetting the Past and Neglecting the Future. Commentary: A Crisis in Comparative Psychology: Where Have All the Undergraduates Gone?

**DOI:** 10.3389/fpsyg.2015.01823

**Published:** 2015-11-27

**Authors:** Marco Vasconcelos, Josefa N. S. Pandeirada

**Affiliations:** ^1^Animal Learning and Behavior Lab, School of Psychology, University of MinhoBraga, Portugal; ^2^Behavioural Ecology Research Group, Department of Zoology, University of OxfordOxford, UK; ^3^CINTESIS, Department of Education, University of AveiroAveiro, Portugal; ^4^Department Psychological Sciences, Purdue UniversityWest Lafayette, IN, USA

**Keywords:** academia, comparative psychology, cross-departmental programs, local contingencies, non-human behavior

The opinion piece published by Abramson (2015) echoes many of the predicaments faced by modern-day comparative psychologists. It is a thoughtful and incisive examination of the current state of our discipline and a welcome invitation for action. This commentary is not intended to question either the *diagnosis* or the *treatment* proposed. Instead, it should be read as additional food for thought.

Abramson (2015) clearly identifies the major problem faced by comparative psychology: to find the “next generation of comparative psychology students” (p. 1). We agree that the field has become less appealing to students, but the main problem is upstream: We do not need a new generation of students if there are no professors to teach them. In other words, our worries are with the current (and past) generation of graduate students struggling to find stable academic positions. Although this problem cuts across disciplines, purely behavior-based animal research has disappeared from the bulk of job offers as acknowledged by Abramson. Whenever animal research is mentioned, it is in the context of understanding the neural underpinnings of some behavioral or cognitive process or in the psychopharmacology realm. These are noble research domains, but few comparative psychologists would be truly competitive in such specialized fields. This indeed threatens the very existence of the discipline.

Abramson (2015) proposes that we should make clear to students that comparative psychology encompasses human behavior. This will certainly attract students but the next step may not be as easy to assimilate. The belief in human uniqueness is perhaps one of the reasons why psychology undergraduate programs are so popular. Nonetheless, one of our main commitments as scientists is to scientific integrity and that often entails reducing seemingly complex human and non-human cognitive feats to simple mechanisms frequently shared across taxa—the now proverbial killjoy explanations (e.g., Shettleworth, 2010). Humans certainly have unique cognitive aptitudes but they are best viewed as an assortment of species-specific, species-general, and domain-general processes. This will disappoint some and inspire others. We want to keep at least the latter.

So, how do we inspire? There is no obvious answer, but nothing is more compelling than practicals. A series of well organized, self-contained hands-on lab experiments focusing on key concepts and techniques are usually the sparkle students need. Also, we should never forget what we have accomplished so far. Sometimes it is easy to forget that much of what we now know about cognition and behavior came from highly original comparative (or “proto-comparative”) research. A small sample of pioneering research includes Darwin's contributions that shed new light on the concepts of instinct (Darwin, 1859) and emotion (Darwin, 1872); the pioneering experiments of Pavlov (1927) on conditional reflexes and those of Skinner (1938) on operant behavior that established the most basic units of animal and human learning; the Garcia and collaborators experiments (e.g., Garcia and Koelling, 1966) on taste aversion that showed how the range of what can be learned is constrained by the biology and ecology of the organism under study; the experiments of Herrnstein (e.g., Herrnstein and Loveland, 1964) on the formation of concepts, that illustrate how little we still know about cognition. We are at least part of the proud followers of this (surely heterogeneous) tradition and should impart it to our students.

Concerning the scope of comparative psychology, Abramson (2015) suggests that we should convey to students that “the research of many ‘animal psychologists’ clearly makes explicit that their work is designed to be integrated with human behavior” (p. 2). Even though we acknowledge that this may attract students, we are not among those assuming that such integration is either essential or should necessarily be conveyed to students. Comparative psychology will only harm and constrain itself by limiting its scope to cases of palpable integration among species. By doing so, we will turn our back on a plethora of provocative behavioral phenomena. Consider, for example, non-neuronal organisms: Are they excluded from our studies by definition? (for an enlightening review, see Reid et al., 2015). Our research programs should originate from a sense of wonder and enthusiasm for the unknown—and enthusiasm attracts students.

Finally, as Abramson (2015) acknowledges, comparative psychology requires training in a diversity of fields, from animal behavior, learning, and computer programming, to statistics, behavioral genetics, and evolutionary theory, among many others. Such a graduate program would be difficult to implement in almost any department. We are used to thinking of our departments as almost self-contained, but students will certainly benefit from interdisciplinary training programs that foster research and graduate training across disciplines (probably, with psychology and biology at the forefront). Besides training in different topics, students would avoid frequent misconceptions of other fields (e.g., the common confusion between proximate and ultimate explanations in human evolutionary literature; Scott-Phillips et al., 2011) and learn that different fields approach the same problems with different lenses and give us exactly what we need: Answers to different questions about the same phenomena.

All things considered, the absence of graduate programs specifically called Comparative Psychology is not worrisome in our view. Given the scope of the field, cross-departmental programs have more benefits than drawbacks. On the contrary, the reduced number of undergraduate courses in Comparative Psychology and particularly the lack of recognition of its existence in introductory psychology textbooks, as noted by Abramson (2015), is disturbing because this is where fascination should start. Perhaps we pretentiously assume that the relevance of our work is self-evident to our colleagues but this does not fully explain the current state of affairs. Regrettably, this takes us back to the starting point of this commentary: Comparative psychologists are not being absorbed into academia. Those of us retiring are, more often than not, the last comparative psychologist in our department. This does not reflect the state of comparative psychology, but the state of psychology itself: An immature science shouldering for recognition among the “sharks” of science, responding to local contingencies and forgetting its own future.

## Author contributions

MV and JNSP jointly wrote the manuscript.

### Conflict of interest statement

The authors declare that the research was conducted in the absence of any commercial or financial relationships that could be construed as a potential conflict of interest.
